# Rewiring local immune milieu by boosting dendritic cell for pancreatic cancer treatment

**DOI:** 10.1038/s41392-020-00225-4

**Published:** 2020-07-02

**Authors:** Yifan Zhang, Qun-Ying Lei

**Affiliations:** 1 Fudan University Shanghai Cancer Center & Institutes of Biomedical Sciences; Cancer Institutes; Key Laboratory of Breast Cancer in Shanghai; The Shanghai Key Laboratory of Medical Epigenetics, Shanghai Medical College, Fudan University, Shanghai, China; 2 Department of Oncology, Shanghai Medical College, Fudan University, Shanghai, China; 3 State Key Laboratory of Medical Neurobiology, Fudan University, Shanghai, China

**Keywords:** Cell biology, Tumour immunology

**A recent article by Hedge et al. (Cancer Cell 37, 289–307.e289, 2020) demonstrated that decreased dendritic cell and elevated Th17 cell could explain the immunosuppressive environment of PDAC when compared with that in lung carcinoma. Boosting DC population using Flt3L and CD40 agonist showed improved efficacy on PDAC mouse models and shed new light on its immunotherapeutic strategies.**

**Fig. 1:**
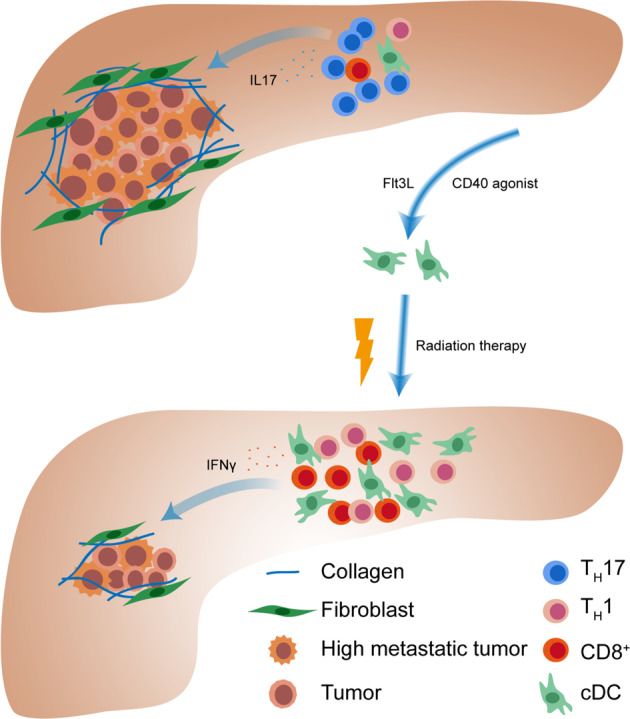
Pancreatic cancer microenvironment has long been known to be immunosuppressive, however, the immune responses to neoantigen expression remain elusive. Hedge et al. engineered a neoantigen-expressing PDAC mouse model and revealed the cDC-scarce T_H_17-abundant immune milieu. Boosting cDCs infiltration using Flt3L and CD40 agonist could significantly increase intratumoral CD8^+^ and T_H_1 cell number and extend survival in combination with radiation therapy

Pancreatic ductal adenocarcinoma (PDAC) is among the leading causes of cancer-induced mortality, and the overall prognosis remains poor despite of the growing understanding about it.^1,2^ Immunotherapy has achieved many successes for multiple cancer treatments like non-small-cell lung cancer and melanoma cancer.^3^ Unfortunately, PDAC remains refractory to immunotherapy largely due to its immunosuppressive environment.^2^ Now a new strategy has been proved to rewire the local immune milieu and mobilize anti-tumor immune cells into pancreas to kill cancer cells^1^ (Fig. 1).

One basic principle of immunotherapy is that tumor cells that produce neoantigen (mainly encoded by tumor specific mutations) could be recognized and destroyed by immune cells.^3^ However, it is formidable to systemically analyze the interactions between neoantigens and immune cells during de novo pancreas cancer progression due to limited tumor specific neoantigens expression.^4^ Hedge et al.^1^ elegantly engineered a mouse model and tried to investigate how neoantigen shapes anti-tumor immunity and affects PDAC progression.

Hedge and colleagues engineered a mouse expressing a model neoantigen OVA (ovalbumin) under the control of *Cre* activation and crossed it with the widely used PDAC mouse model KPC (*Kras*^*LSL-G12D*^; *Trp53*^*fl/fl*^; *p48-Cre*) to generate KPC-OG mice. First, they proved that OVA expressed on tumor metaplasia and colocalized with pancreatic ductal cell marker cytokeratin 19^+^ (CK19). Then they confirmed OVA could be functionally presented using OVA-specific OT-I CD8^+^ T cells. They also confirmed that endogenous OVA-specific T cells were not cleared by thymic deletion and these cell population were increased in pancreas at early pancreatic lesions. These data demonstrated that KPC-OG mouse was a reliable tool to investigate the antigen-specific response under pancreatic cancer context.

The next part of experiment was to determine the physiological outcome of neoantigen expression. Surprisingly, in contrary to KP lung mouse models that OVA neoantigen could boost anti-tumor immune response, OVA expression in KPC mice induced accelerated tumor progression, higher metastasis, and reduced survival. Checkpoint immunotherapy did not show improved efficacy in KPC-OG mice. These phenotypes indicated that neoantigen expression induced a pathogenic pro-tumor immune response. Indeed, CD4^+^ T cell depletion decreased tumor burden in KPC-OG mice, and further analysis demonstrated that IL17-secreting T_H_17 cell was increased in its pancreas and had a key role in mediating tissue fibrosis and promoting tumor progression. To answer which factor determines the different adaptive immune responses to neoantigen between lung and pancreatic cancer, the authors performed immune profiling experiment and found that conventional dendritic cells (cDCs) significantly decreased in pancreas in KPC-OG mice. They also confirmed this phenomenon in human PDAC tissue. cDC is an important innate immune cell population that could represent neoantigen and migrate to tumor draining lymph node (dLN) to activate anti-tumor T cell responses. Consequently, pancreas dLNs showed almost no migratory cDCs and far fewer OVA-specific CD8^+^ T cells when compared with same-stage lung dLNs.

The above data indicated that cDCs could be a crucial determinant to boost the anti-tumor immunity in pancreas. The authors next sought to repress PDAC progression through increasing cDCs number. At early stage of tumorigenesis, the usage of Fms-related tyrosine kinase 3 ligand (Flt3L), which could promote cDCs hematopoietic mobilization, reduced pro-tumor T_H_17 cells and promoted anti-tumor CD8^+^ CTL numbers. However, in mice bearing established pancreatic tumors, administration of Flt3L showed weak effect on T cell response due to ineffective DC mobilization. Therefore, Hedge et al. attempted a strategy to both mobilize and activate DC cells using Flt3L and CD40 or STING agonist on established PDAC mice and this treatment was more effective. Compared with single treatment, combined treatment triggered significantly increased cDCs, CD8^+^ CTL, CD4^+^ T_H_ cell, as well as NK, NKT, and γδT cell infiltration, decreased collagen deposition, and desmoplastic α-SMA^+^ fibroblasts. In order to get better efficacy, the authors used radiation therapy (RT), which could induce massive cell death and activate immune response, in combination with Flt3L and CD40 agonist to treat PDAC mice and found reduced tumor burden and higher survival rate over RT monotherapy.

Collectively, through comprehensive analysis of different engineered mouse models, Hedge and colleagues proved that cDCs–T_H_17 axis is the most dysregulated immune response after neoantigen expression in PDAC compared with lung tumor microenvironment, and boosting cDCs recruitment and activation using Flt3L and CD40 agonist could improve efficacy in combination with RT. Previous clinical analysis has demonstrated that the coexistence of neoantigen and CD8^+^ T cell correlated with longer survival rate of PDAC patients^5^ and highlights the importance to understand the neoantigen-mediated immune milieu for this checkpoint blockade-refractory cancer treatment. Considering that clinical trials with Flt3L and CD40 agonist have been tested for multiple solid tumors, this research provides experimental evidence and clinical basis for DC-targeted immune therapy against pancreatic cancer treatments. Furthermore, other therapies could take account of the DC-depleted immune milieu during pancreatic cancer progression for better strategies.
